# Robust CRISPR-Cas9 Genetic Editing of Primary Chronic Lymphocytic Leukemia and Mantle Cell Lymphoma Cells

**DOI:** 10.1097/HS9.0000000000000909

**Published:** 2023-06-07

**Authors:** Judith Mateos-Jaimez, Maurizio Mangolini, Anna Vidal, Marta Kulis, Dolors Colomer, Elias Campo, Ingo Ringshausen, Jose I. Martin-Subero, Alba Maiques-Diaz

**Affiliations:** 1 Institut d’Investigacions Biomèdiques August Pi i Sunyer (IDIBAPS), Barcelona, Spain; 2 Department of Hematology and Cambridge Stem Cell Institute, University of Cambridge, United Kingdom; 3 Centro de Investigación Biomédica en Red de Cáncer (CIBERONC), Madrid, Spain; 4 Hematopathology Unit, Pathology Department, Hospital Clinic, Barcelona, Spain; 5 University of Barcelona, Spain; 6 Institució Catalana de Recerca i Estudis Avançats (ICREA), Barcelona, Spain

Chronic lymphocytic leukemia (CLL) and mantle cell lymphoma (MCL) are 2 lymphoid neoplasms characterized by the proliferation and accumulation of mature small CD5+ B cells, commonly involving bone marrow, blood, and lymphoid organs.^1^ CLL is considered an indolent disease, whereas the clinical course of the majority of MCL patients is more aggressive. However, the clinical evolution of both malignancies is very heterogeneous. This heterogeneity is exemplified by 2 major clinico-biological subtypes described in both diseases, which are characterized by the level of somatic hypermutation of the immunoglobin heavy chain variable (IGHV) genes. In CLL, those cases harboring unmutated IGHV genes (U-CLL) are derived from germinal center unexperienced cells and show a more aggressive clinical behavior than those carrying mutated IGHV genes (M-CLL), which stem from germinal center-experienced cells. In MCL, the translocation t(11;14) is common to both MCL subtypes, leading to cyclin D1 overexpression. The most common and clinically aggressive subtype is called conventional MCL (cMCL) and derived from mature pregerminal center B cells, carrying no or limited IGHV mutations. Nonnodal MCL (nnMCL) is less common and an indolent subtype, derived from germinal center-experienced cells and carries a higher load of IGHV mutations.^1^ Over the last decade, the landscape of the genomic, epigenomic, and transcriptional features of CLL and MCL has been described in several pivotal studies, highlighting the coincidences and differences between the aggressive and indolent subtypes of both diseases.^2,3^ While the prognostic significance of some of these alterations is known, their specific contributions to disease pathogenesis remains largely unexplored.

Attempts to study the molecular mechanisms underlying CLL and MCL using primary cells have encountered several challenges, mostly owing to the importance of the tumor microenvironment factors for the survival and proliferation of the malignant B cells. Thus, most experimental settings used for these diseases include genetically modified mouse models for CLL^4^ or xenograft models for MCL,^5^ and human cell lines,^6,7^ which have numerous discrepancies with human primary cells. Importantly, these model systems mostly recapitulate the more aggressive forms of both neoplasms (U-CLL and cMCL), but they are not appropriate tools for studying indolent subtypes of the disease. Thus, there is a need to directly apply molecular methods, such as CRISPR-Cas9 genome editing technology, in primary malignant B cells in vitro as they preserve the clinico-biological spectrum of CLL and MCL. Previous functional studies have been limited by the difficulty of growing CLL and MCL cells ex vivo, as well as their resistance to most gene transfer methods, which have made downstream analysis challenging.^8^ While recently it has been demonstrated that it is possible to genetically manipulate nonactivated B cells,^9^ the use of gene editing tools in malignant B cells is still elusive. Thus, to advance further in the genetic manipulation of primary malignant B cell, such as CLL and MCL, we took advantage of our cell culture system that allows the in vitro expansion of patient-derived CLL/MCL cells for several weeks, regardless of the clinico-biological subtype of the disease.^10^ Using this cell culture system, in which primary cells are exposed to human CD40-ligand, IL21, and BAFF secreted by murine stromal cells, we have now established a robust method for CRISPR-Cas9 editing of patient-derived malignant-activated CLL/MCL cells.

As an initial proof of concept, we targeted the CD19 pan-B cell marker, highly expressed in both CLL and MCL cells. Patient-derived and cryopreserved CLL and MCL cells, isolated from peripheral blood samples, were expanded for 3 days in vitro and subsequently electroporated with optimized conditions (see Suppl. Figure S1 for detailed information) to allow the entrance of the Cas9-CD19 guide RNA (gRNA) complex into the cells (Figure 1A). To assess electroporation efficiency, gRNA was labeled with a fluorescent tracer (ie, ATTO550). Cell viability and electroporation efficiency, measured after 24 hours, demonstrated a remarkable low percentage of dead cells, similar to nonelectroporated cells. With this method, high transfection efficiency was accomplished with over 90% of cells containing the fluorescent tracer (Figure 1B and 1C). We followed the degree of CD19 depletion over time and observed a rapid decrease in protein level especially in CLL, which was already evident at day 2 (Figure 1D and 1E; Suppl. Figure S2D and S2E). Four days after electroporation, CD19 levels were downregulated by >90% in all CLL samples tested (n = 8), and by 75% in MCL samples (n = 4). These low protein levels were maintained over time and did not affect cell viability (Figure 1D and 1E; Suppl. Figure S2D and S2E; Suppl. Figure S3B and S3D). We performed amplicon sequencing to measure the percentage of *CD19* alleles with an insertion/deletion mutation induced by the Cas9-gRNA and observed 64% and 65% of mean allele editing in CLL (n = 3) and MCL (n = 3), respectively. No changes on the proportion of allele editing were found over time (Suppl. Figure S2A-S2C). In summary, we have developed a method for the transient transfection of the Cas9-gRNA complex into patient-derived malignant B cells, without inducing cell death and with highly efficient gene editing and protein depletion.

**Figure 1.**
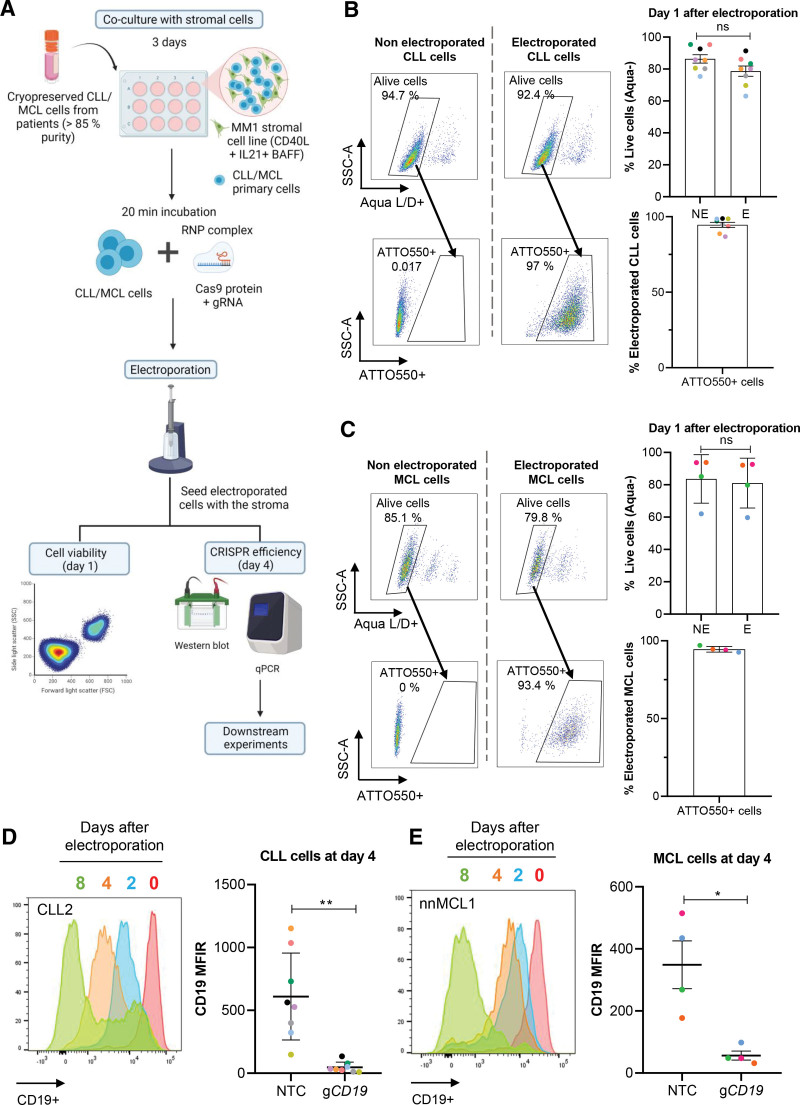
**Efficient CRISPR-Cas9 editing of primary malignant B cells.** (A) Schematic representation of the electroporation-based CRISPR-Cas9 methodology used with CLL and MCL cells. Created with BioRender.com. (B) and (C) FACS panels show percentage of live and successfully transfected cells compared with non electroporated cells at day 1 after electroporation for (B) CLL (n = 8) or (C) MCL (n = 4) primary cells. Upper dot plot and bar graphs shows the percentage of live cells (Aqua−) ± SEM in electroporated (E) vs non electroporated (NE) cells. Lower dot plot and bar graphs shows the percentage of electroporated cells (ATTO550+) ± SEM in the live population. Unpaired *t* test, *P* > 0.05. (D) and (E) Histogram shows CD19 protein levels assessed by flow cytometry at day 0, 2, 4, and 8 after electroporation in 1 exemplary sample of (D) CLL or (E) MCL. Box plot on the right represents CD19 MFIR value in NTC and g*CD19*-electroporated cells at day 4 after electroporation. Paired *t* test, **P < 0.01, *P < 0.05. Each color represents a different case. NTC = nontargeting control; g*CD19* = gRNA targeting *CD19* gene; MFIR = median fluorescence intensity ratio; CLL = chronic lymphocytic leukemia; MCL = mantle cell lymphoma.

To further assess the utility of our method to study the downstream effect of genes involved in CLL or MCL pathogenesis, we targeted 2 important cell cycle regulatory proteins: cyclin D2 (*CCND2*) in CLL and cyclin D1 (*CCND1*) in MCL cells. *CCND1* overexpression is a hallmark of MCL development,^1^ while *CCND2* has been shown to be upregulated in CLL cells, particularly, at the proliferation centers in lymph nodes.^11,12^ Concordantly, *CCND2* becomes highly upregulated in CLL cells exposed to the proproliferative stimuli of our cell culture system (Figure 2A). We first induced *CCND2* depletion using 2 independent gRNAs electroporated with the Cas9 protein into 8 CLL primary cases (3 U-CLL and 5 M-CLL, see Suppl. Table S1 for patient characteristics). At day 4, we observed a 70% reduction of *CCND2* levels, both at the mRNA (Suppl. Figure S3A) and protein level (Figure 2B). Remarkably, cyclin D2 depletion had a major impact on cell proliferation on both U-CLL and M-CLL cases, as by day 2 we observed a mean cell growth reduction of 62% and 50% with each gRNA used (Figure 2C), without significant impact on cell viability (Suppl. Figure S3B). In parallel, *CCND1* gene was targeted with 2 independent gRNAs in 4 MCL primary cases (2 cMCL and 2 nnMCL, see Suppl. Table S2 for patient characteristics). At day 4, we observed >65% reduction of cyclin D1 protein level with both gRNAs (Figure 2D; Suppl. Figure S3C). Interestingly, upon cyclin D1 depletion, cell viability was not significantly affected (Suppl. Figure S3D), but cell proliferation was reduced in cMCL cases (n = 2), while we did not observe significant changes in the nnMCL cases (n = 2) (Figure 2E). Despite the small sample size, this observation suggests that the contribution of cyclin D1 to cell proliferation may differ between the 2 MCL clinico-biological subtypes and reinforces the importance of studying the pathobiology of MCL with primary cells, which preserve the diversity of cMCL and nnMCL cases.

**Figure 2.**
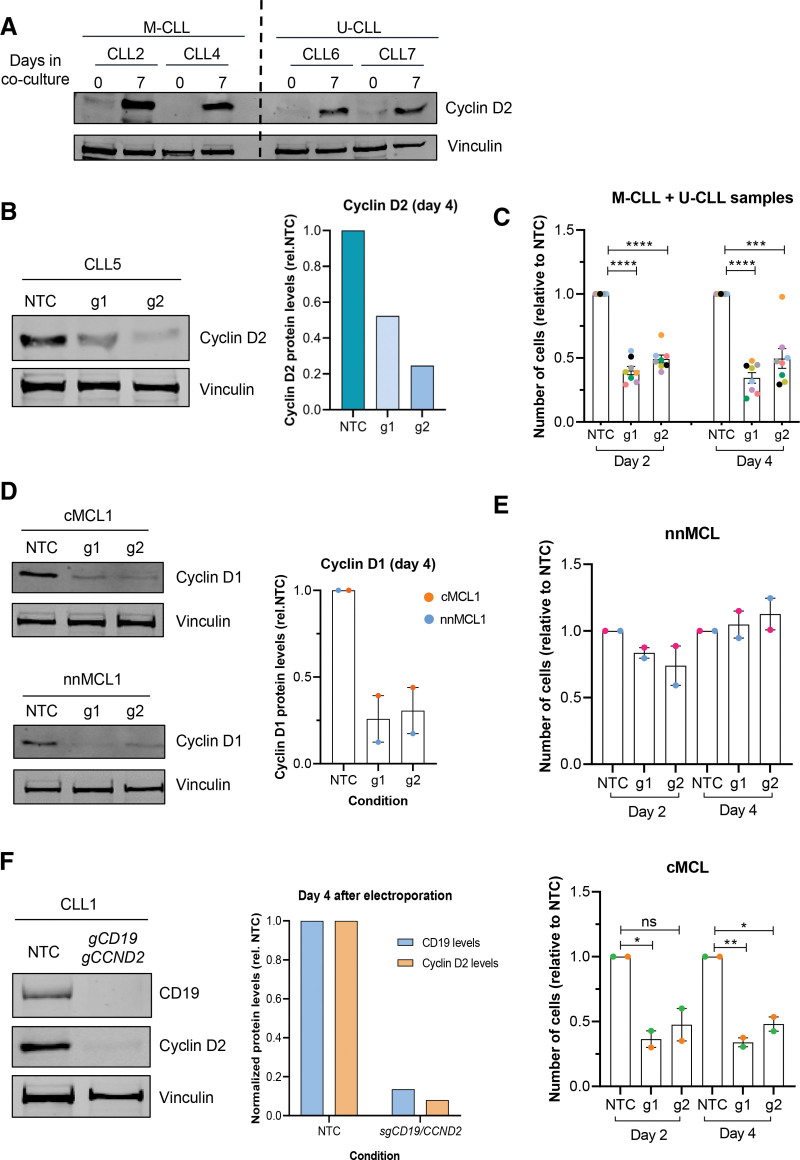
**Single and dual gene editing of cyclin genes impair proliferation of malignant B cells**. (A) Western blot shows cyclin D2 protein levels before and after coculture with MM1 cells for 7 days in CLL cells (2 U-CLL and 2 M-CLL). (B) Western blot shows cyclin D2 protein levels at day 4 in CLL5 upon electroporation in the indicated conditions. Bar plot shows ImageJ protein quantification normalized with NTC (right). (C) Bar plot displays the relative cell number of NTC and *CCND2* g1/g2-electroporated CLL cells at day 2 and day 4 after electroporation ± SEM (n = 8). Unpaired *t* test NTC vs g1/g2, ****P < 0.000. (D) Western blot shows protein levels of cyclin D1 at day 4 in NTC and *CCND1* g1/g2-electroporated cells in 1 case of cMCL and nnMCL. Bar plot shows ImageJ protein quantification ± SEM normalized with NTC in cMCL (orange) and nnMCL (blue). (E) Bar plot displays the relative cell number in NTC and *CCND1* g1/g2-electroporated cells in nnMCL cells (upper) and cMCL (lower) at day 2 and day 4 after electroporation ± SEM (n = 4). Unpaired *t* test NTC vs g1/g2; ns for nnMCL and *P < 0.05 and **P < 0.01 for cMCL. (F) Western blot shows CD19 and cyclin D2 protein levels in NTC or double-edited CLL cells, sorted by CD19- in CLL1. Bar plot shows ImageJ protein quantification normalized with NTC for CD19 levels (blue) and Cyclin D2 (orange). Each color represents a different case. M-CLL = IGHV mutated cases; U-CLL = IGHV unmutated cases; NTC = nontargeting control; g1/g2 = 2 independent gRNAs targeting *CCND2* or *CCND1* genes; cMCL = conventional MCL; nnMCL = nonnodal MCL; CLL = chronic lymphocytic leukemia; MCL = mantle cell lymphoma.

Finally, we tested the efficiency of the simultaneous electroporation of 2 gRNAs to induce dual target editing. For this, *CD19* and *CCND2* gRNAs were combined with a Cas9 protein and subsequently electroporated into CLL cells. We observed that cell viability was not affected and that 60% of the cell population contained both gRNAs, each labeled with a different fluorescent tracer (n = 2) (Suppl. Figure S4A and S4B). At day 4, we sorted double gRNA transfected CLL cells by CD19−, and confirmed that this method achieved an almost complete depletion of both CD19 and cyclin D2 proteins (Figure 2F; Suppl. Figure S4C and S4D). Importantly, the levels of protein depletion were similar to those obtained with single gRNAs, indicating that our method of dual gene targeting is feasible and highly effective (Suppl. Figure S4C).

Together, these results demonstrate that our method of transient CRISPR-Cas9 editing is highly efficient in modifying even >1 gene at once in malignant B cells derived from patients. Our protocol opens the door to deepen scientific investigations into the biology of malignant B cells using CRISPR-Cas9 methodology directly in primary patient samples, and overcomes the current dependency on cell lines or murine models^13–15^ to study these diseases. In contrast to a recently published protocol to induce mRNA-based gene expression and gene editing in normal B cells,^9^ we focused on developing an efficient CRISPR-Cas9 editing method specifically for CLL and MCL samples. Notably, we observed that prestimulated malignant cells showed greater electroporation efficiency than unstimulated cells (Suppl. Figure S1), indicating a difference to normal B cells. On the basis of our data, proliferation of CLL/MCL cells seems to be key for a successful and efficient gene editing. Moreover, using our cell coculture method,^10^ we have shown a permanent protein depletion that is maintained in any offspring cell, enabling longer follow-up downstream analyses and targeting of several candidate genes simultaneously.

In summary, we provide a robust and efficient gene editing method of primary human malignant B cells for the scientific community. Importantly, this method complements our protocol to stably transduce primary cells,^10^ allowing both depletion and overexpression of genes of interest in primary CLL/MCL cells, to study the differences underlying their clinico-biological diversity.

## ACKNOWLEDGMENTS

We thank the IDIBAPS Flow Cytometry and Cell Sorting core facility for their help.

## AUTHOR CONTRIBUTIONS

JM-J preformed and analyzed experiments and wrote the article. AV and MK performed experiments. MM performed experiments and provided key reagents. DC and EC provided the human primary sample collections and their biological and clinical annotation. AM-D directed and analyzed experiments. This project was designed by IR, JIM-S, and AM-D, and all three wrote the article. All authors reviewed the final version.

## DISCLOSURES

The authors have no conflicts of interests to disclose.

## SOURCES OF FUNDING

This work was supported by research funding from the Spanish Ministry of Science and Innovation (PID2020-118167RB-I00), Fundació La Marató de TV3 (201924-30), the European Research Council (ERC) under the European Union’s Horizon 2020 research and innovation programme (810287, BCLLatlas), and CIBERONC (CB16/12/00225 and CB16/12/00334), the Beatriu de Pinós Programme of the Generalitat de Catalunya to AM-D (AGAUR 2018-BP-00231), and “La Caixa” Foundation to JM-J (LCF/BQ/DR20/11790011). This work was also funded by Cancer Research UK (CRUK; C49940/A17480-IR was a senior CRUK fellow) and Kay Kendall Leukaemia Fund (MM-KKL1258). This work was developed at the Center Esther Koplowitz (CEK, Barcelona, Spain).

## Supplementary Material


