# Supplementary material for: Robust CRISPR-Cas9 Genetic Editing of Primary Chronic Lymphocytic Leukemia and Mantle Cell Lymphoma Cells
Source: Hemasphere. 2023 Jun 7;7(6):e909. doi: 10.1097/HS9.0000000000000909 (PMC10249715; doi:10.1097/HS9.0000000000000909)
Supplement: Supplementary file 1 [file hs9-7-e909-s001.docx]

**Supplemental Material**

**Robust CRISPR-Cas9 genetic editing of primary chronic lymphocytic leukemia and mantle cell lymphoma cells**

Judith Mateos-Jaimez^1*^, Maurizio Mangolini^2*^, Anna Vidal^1^, Marta Kulis^1^, Dolors Colomer^1,3,4,5^, Elias Campo^1,3,4,5^, Ingo Ringshausen^2, †^, Jose I. Martin-Subero^1,3,6, †^ and Alba Maiques-Diaz^1,†^.

**
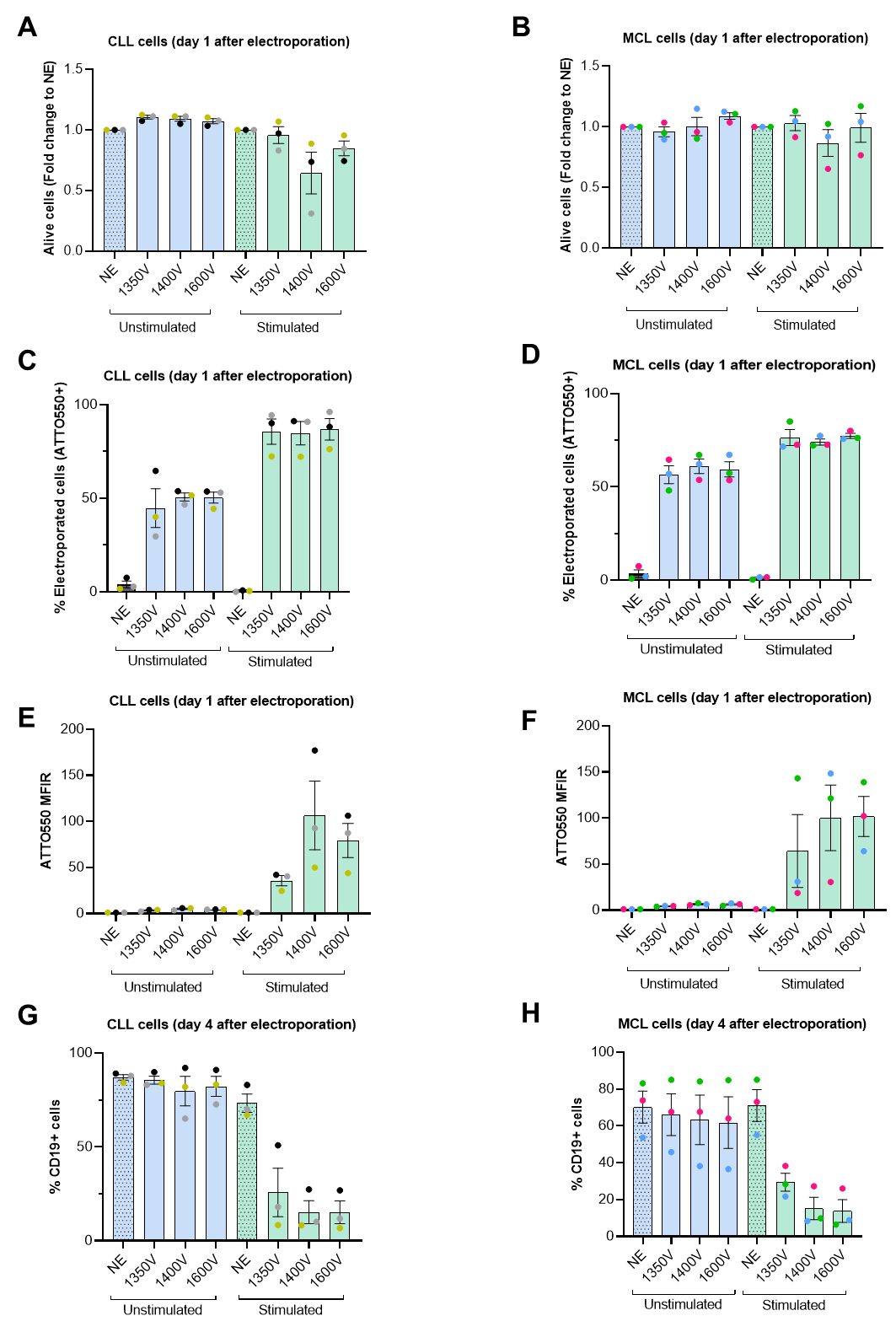
Supplementary Figure 1. Optimization of the CRISPR-Cas9 protocol.** Three independent CLL or MCL cryopreserved samples were exposed to the indicated electroporation conditions using the Neon Transfection system. All cases were electroporated directly from the cryopreservation (blue bars) or upon three days of stimulation with the MM1 stroma (green bars). (A-B) Bar graphs show the percentage of live cells (Aqua-) ± SEM in electroporated with the indicated conditions versus non-electroporated (NE) cells. (C-D) Bar graphs show the percentage of electroporated cells (ATTO550+) ± SEM in the live population. (E-F) Bar graphs show the ATTO550+ mean fluorescence intensity ratio (MFIR) ± SEM in the live population. (G-H) Bar graphs show the percentage of CD19+ cells in the indicated conditions at day 4 after electroporation. Each color represents a different case.


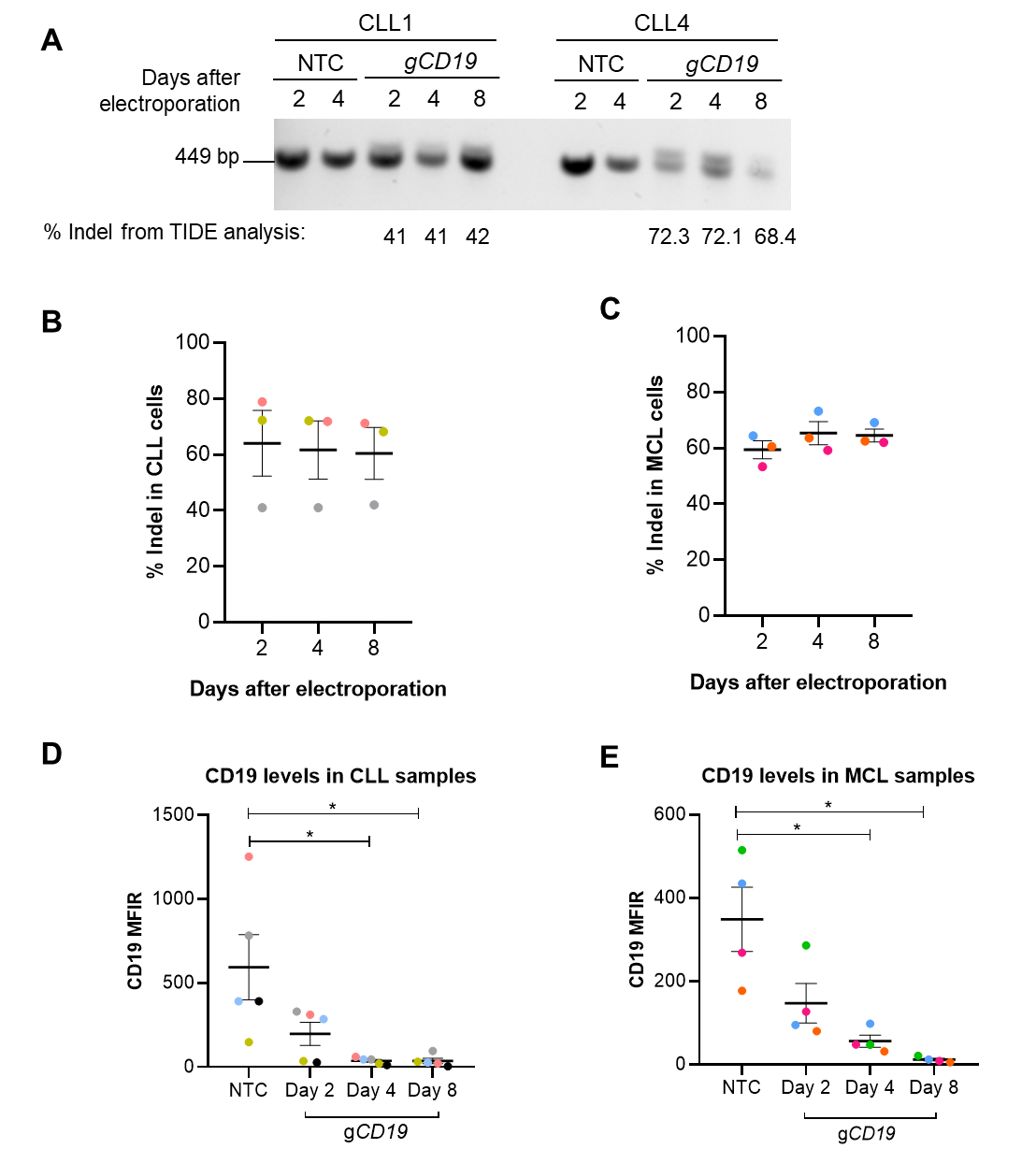


**Supplementary Figure 2. *CD19* gene editing in CLL and MCL primary cells.** (A) Gel shows the *CD19* amplicon covering the *CD19* gRNA target sequence in two individual CLL cases (CLL1 and CLL4). Two DNA bands are only detected at the g*CD19*-transfected CLL cells over time. The lower band corresponds to homoduplex DNA (WT/WT or KO/KO) and the upper slow-migrating band to the heteroduplex WT/KO formation. (B-C). Box plot shows the percentage of edited cells (indel) in 3 cases of CLL (B) or MCL detected by TIDE analysis (D-E) CD19 MFIR values of NTC and *CD19*-edited cells at day 2, 4 and 8 after electroporation in CLL (n=5) (D) and MCL (n=4) (E) samples. Each color represents a different case of CLL/MCL. Paired t-test, *p-val < 0.05. NTC = non-targeting control; g*CD19* = gRNA targeting *CD19.*


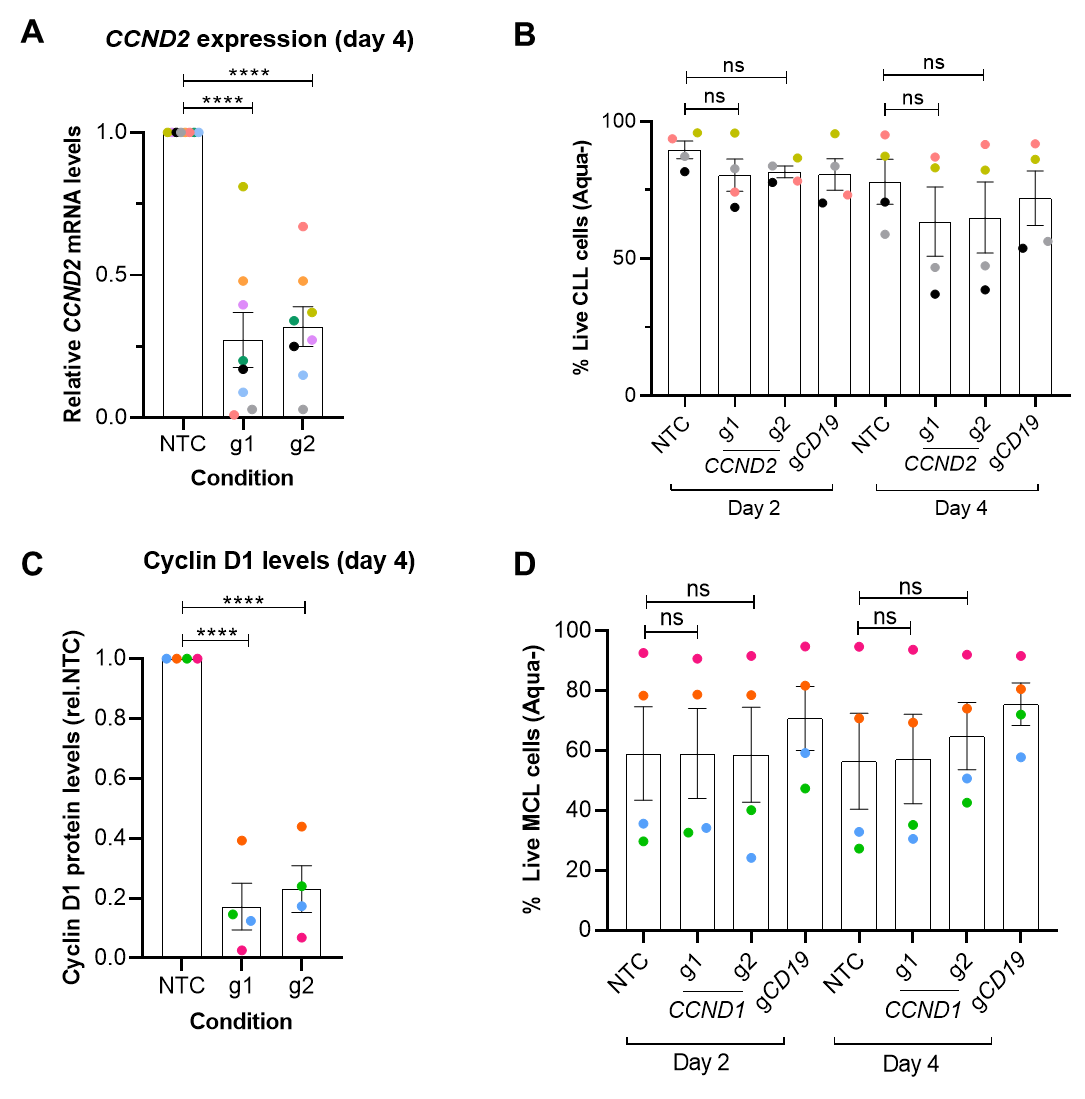


**Supplementary Figure 3. *CCND2* and *CCND1* gene editing in CLL and MCL primary cells.** (A) Bar plot shows the relative levels of *CCND2* mRNA ± SEM in NTC and *CCND2* g1/g2-electroporated CLL cells at day 4 after electroporation (n=8). Unpaired t-test, ****p-val < 0.0001. (B) Bar plot shows percentage of live cells (Aqua-) ± SEM at day 2 and day 4 in NTC and *CCND2* g1/g2 or g*CD19*-electroporated CLL cells (n=4). Paired t-test, ns, p-val > 0.05. (C) Bar plot shows ImageJ protein quantification of cyclin D1 levels normalized with NTC ± SEM of 4 independent MCL cases. Unpaired t-test, ****p-val < 0.0001. (D) Bar plot shows percentage of live cells ± SEM at day 2 and day 4 in NTC and *CCND1* g1/g2 or g*CD19*-electroporated MCL cells (n=4). Paired t-test, ns, p-val > 0.05. Each color represents a different case. NTC = non-targeting control; g1/g2 = two independent gRNAs targeting *CCND2* or *CCND1* genes.


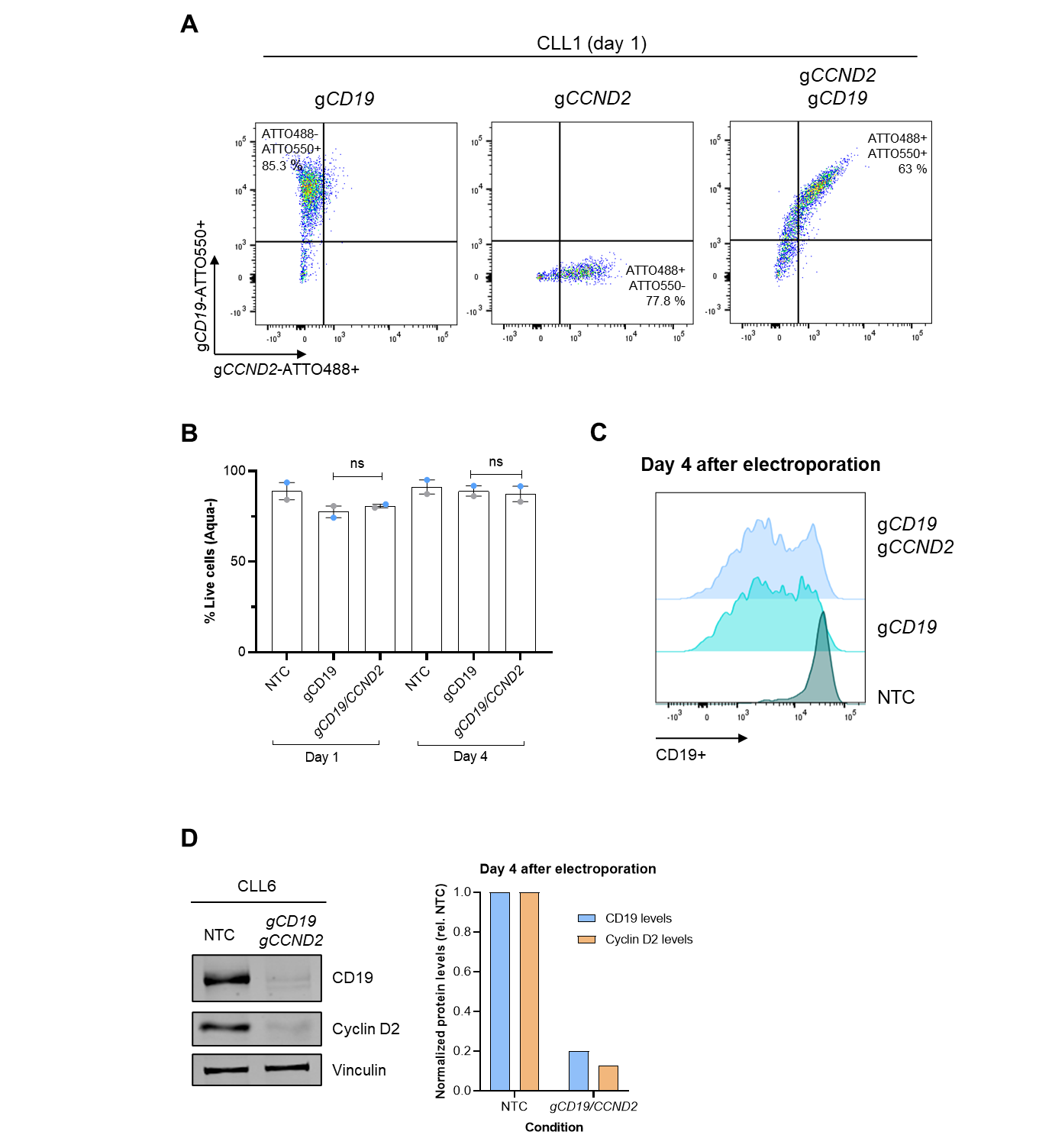


**Supplementary Figure 4. Dual editing of *CD19* and *CCND2* genes in CLL primary cells.** (A) FACS plots show the percentage of electroporated cells with one single gRNA (containing g*CD19*-ATTO550 or g*CCND2*-ATTO488) and two gRNA (g*CD19*-ATTO550 + g*CCND2*-ATTO488) in CLL1. (B) Bar graphs show the percentage of live cells (Aqua-) ± SEM in electroporated cells with the indicated conditions. (C) Western blot shows CD19 and cyclin D2 protein levels in NTC or double-edited CLL cells, sorted by CD19- in CLL6. Bar plot shows ImageJ protein quantification normalized with NTC for CD19 levels (orange) and Cyclin D2 (blue). (D) Histogram displays CD19 protein levels assessed by flow cytometry at day 4 in NTC, single edited g*CD19* cells and double edited g*CD19*-g*CCND2* in sample CLL6. NTC = non-targeting control; g*CD19*/*CCND2* = gRNA targeting each gene of interest.
