# Supplementary material for: Robust CRISPR-Cas9 Genetic Editing of Primary Chronic Lymphocytic Leukemia and Mantle Cell Lymphoma Cells
Source: Hemasphere. 2023 Jun 7;7(6):e909. doi: 10.1097/HS9.0000000000000909 (PMC10249715; doi:10.1097/HS9.0000000000000909)
Supplement: Supplementary file 2 [file hs9-7-e909-s002.docx]

**Supplementary Table 1**. Characteristics of CLL samples used in the study.

| **CLL case** | **IGHV status** | **IGHV % identity to germline** | **IGHV gene** | **Gender** | **Age at diagnosis** | **Color in figures** |
| --- | --- | --- | --- | --- | --- | --- |
| 1 | MUT | 97.25 | IGHV4-61 | M | 45 |  |
| 2 | MUT | 89.58 | IGHV3-30 | M | 60 |  |
| 3 | MUT | 93.87 | IGHV3-49 | M | 58 |  |
| 4 | MUT | 97.19 | IGHV4-34 | F | 64 |  |
| 5 | MUT | 94.09 | IGHV1-8 | M | 48 |  |
| 6 | UNMUT | 100 | IGHV1-69 | F | 59 |  |
| 7 | UNMUT | 98.61 | IGHV3-30 | M | 45 |  |
| 8 | UNMUT | 100 | IGHV1-2 | M | 62 |  |

**Supplementary Table 2.** Characteristics of MCL samples used in the study.

| **MCL case** | **IGHV status** | **IGHV % identity to germline** | **SOX11** | **Gender** | **Age at diagnosis** | **Color in figures** |
| --- | --- | --- | --- | --- | --- | --- |
| nnMCL1 | MUT | 95.14 | Negative | M | 75 |  |
| nnMCL2 | MUT | 97.22 | Negative | M | 80 |  |
| cMCL1 | UNMUT | 97.92 | Positive | M | 76 |  |
| cMCL2 | UNMUT | 99.65 | Positive | M | 43 |  |

*F: female; M: male*

**Supplementary material and methods**

**Primary cells and cell culture**

After patients’ informed consent and in accordance with the Helsinki Declaration, peripheral blood was obtained from patients with a diagnosis of CLL or MCL before any treatment was given. The study was approved by the clinical research ethics committee of the Hospital Clinic of Barcelona. CLL and MCL cells were obtained from cryopreserved peripheral blood mononuclear cells. All samples used in this study had a purity > 85% that was assessed by immunostaining of CD19, CD20, CD5 and CD45 followed by flow cytometry. After thawing, cells were cultured with MM1 feeder cells^1^ in Advanced Roswell Park Memorial Institute medium (Advanced RPMI-1640; Invitrogen, Carlsbad, CA) with GlutaMAX containing 10% FBS (Gibco), 100 IU/ml penicillin and 100µg/ml streptomycin and kept at 37°C in a humidified incubator (5% CO_2_ and 95% atmosphere). Primary cells were cultured with MM1 cells for 3 or 4 days before electroporation.

MM1 feeder cells (bone marrow-derived murine stromal cell line) were cultured in MEM Alpha+GlutaMAX medium (ThermoFisher Scientific, Waltham, MA, USA) supplemented with 10% FBS (Gibco), 10% horse serum (Sigma-Aldrich, Dorset, UK), 10 μM 2-Mercaptoethanol and 1% penicillin/streptomycin (Gibco). Feeder cells were seeded in a 12 multi-well plated coated with 0.1% Gelatin 24 hours before the addition of primary CLL/MCL cells at a concentration of 10^6^ cells/ml.

**gRNAs, Cas9 and electroporation**

Guide RNAs, IDT Electroporation Enhancer 100 µM (#1075915) and Cas9 62 µM (#1081058) were purchased from Integrated DNA Technologies (IDT, Coralville, IA, USA). One single gRNA per *CD19* gene and two gRNA per *CCND2* and *CCND1* were employed. Guide RNA was composed by the annealing of crRNA and tracrRNA-ATTO550/ATTO488 (#1075928 and #10007810, IDT). crRNA and tracrRNA were mixed at equimolar concentrations in a sterile microcentrifuge tube to a final concentration of 44 µM, heated at 95°C for 5 min and cooled down at room temperature (RT) (15-25°C). Electroporation transfection was performed on the Neon Transfection System (Thermofisher Scientific). Prior to transfection 0.5 µl gRNA (40 µM), 2 µl IDT Electroporation enhancer (10.8 µM) and 0.5 µl Cas9 (36 µM) were mixed and incubated for 20 min at RT to allow the formation of the ribonucleoprotein complex (RNP). For double gRNAs transfection experiments, 0.5 µl g*CD19* (36 µM), 0.5 µl *CCND2* g1, 2 µl IDT Electroporation enhancer (10.8 µM) and 1 µl Cas9 (36 µM) were mixed and incubated for 20 min at RT. Final molar ratio of RNP complexes is 1:1 gRNA:Cas9. These volumes of gRNA, Cas9 and electroporation enhancer can be used in the range of 0.3-1·10^6^ cells.

Primary cells were counted, washed with PBS and resuspended in Buffer R from the Neon Electroporation System kit (10 µl of Buffer R per 500 000 cells). Then, 500 000 cells in 10 µl of Buffer R were added to the RNP complex and electroporated in the Neon system with the 10-ul pipette tip. To establish the most optimal electroporation condition, three different conditions were tested in unstimulated and stimulated primary CLL/MCL cells:

1. 1350 Volts, 30 ms, 1 pulse
2. 1400 Volts, 20 ms, 3 pulses
3. 1600 Volts, 10 ms, 3 pulses

Upon thawing, unstimulated CLL/MCL cells were left on the incubator with the complemented RPMI medium for 30 min to recover from cryopreservation and then, they were electroporated with the three different conditions. On the other hand, stimulated cells were electroporated after being co-cultured with MM1 cells for 3 days. The balance between highly efficient electroporation and editing efficiency and good cell viability was accomplished with condition 3 (1600 V 10 ms 3 pulses) in stimulated cells (Supplementary Fig. 1). Thus, all the experiments were carried out with stimulated CLL/MCL cells with condition 1600 V 10 ms 3 pulses.

Electroporated cells were immediately transferred to a pre-warmed 12-well plate coated with MM1 cells and maintained until day 4 or 8 after electroporation.

The sequences (5’-3’) of the gRNAs used for each target gene are:

NTC*:* AAAATAGCAGTAAACTCAAC

*CD19:* CGAGGAACCTCTAGTGGTGA ^2^

*CCND1* g1*:* CGTGCCTCCGTAGGTCTGCG

*CCND1* g2*:* GTGTTCAATGAAATCGTGCG

*CCND2* g1*:* ATGTGCTCAATGAAGTCATG

*CCND2* g2*:* CACTTGAAGTAGGAGCACTG

**Flow cytometry**

Cells were stained at 4°C for 20 min with the human CD19-APC (1:100, Clone SJ25C, BD Biosciences, San Jose, CA, USA) and LIVE/DEAD Fixable Aqua Dead Cell Stain (1:300, Thermofisher Scientific) to assess the percentage of alive (Aqua-) and dead cells (Aqua+) in NTC and gene edited cells. Efficiency of electroporation was determined by the percentage of intracellular ATTO550+ or ATTO488+ alive cells that had incorporated the RNPs complexes at day 1 after electroporation. Samples were acquired on a LSR II Fortessa 4L cell analyzer (BD Biosciences) and analyzed using FlowJo v10 software (BD Biosciences). FACS cell sorting was performed using the FACS Aria SORP (BD Biosciences).

**TIDE analysis of gene editing**

Genomic DNA from NTC and *CD19*-edited CLL and MCL cells was collected at day 2, 4 and 8 after transfection and extracted using the QuickExtract™ DNA Extraction Solution (Lucigen #QE0905T, Heidelberg, Germany). A PCR from the extracted DNA using the Kapa HiFi HotStart Ready Mix (Roche #7958927001, Basel, Switzerland) was carried out with primer pairs spanning the targets sites at the *CD19* gene. Amplified DNA was subsequently Sanger sequenced. Edited and control sequences were then analyzed using the TIDE analysis tool^3^ to determine the percentage of insertion/deletion (indel) mutations found in the DNA edited pools. The designed primers are listed below (5’-3’):

*CD19* forward: GCAGACACCCATGGTTGAGTG

*CD19* reverse: CAGCGTTATCTCCCTCTGTGGA

**Expression analysis/RT-qPCR**

Total RNA was extracted using Trizol (Thermofisher) and complementary DNA (cDNA) was obtained using the qScriptTM cDNA SuperMix kit (QuantaBio, Beverly, MA, USA). Quantitative reverse-transcription polymerase chain reaction (RT-qPCR) was performed on isolated mRNA using the fast SYBR reagents and the CFX96 Touch Real-Time PCR Detection System (Biorad, Hercules, CA, USA). Target gene (*CCND2*) expression levels were normalized to *GAPDH* and values are represented as fold change relative to control using the ΔΔCt method. The human designed primers are listed below (5’-3’):

*CCND2 forward:* CCTCCAAACTCAAAGAGACCAG

*CCND2 reverse:* TTCCACTTCAACTTCCCCAG

*GAPDH forward:* CAAGATCATCAGCAATGCCT

*GAPDH reverse:* AGGGATGATGTTCTGGAGAG

**Western blot**

Cultured cells were collected and lysed with RIPA buffer (50mM HEPES pH 7.6, 1mM EDTA, 0.7% Na deoxycholate, 1% NP-40, 0.5M LiCl) complemented with protease inhibitors (Complete EDTA-free tablets, Roche, Basel, Switzerland). A total of 20 µg protein was separated by SDS-PAGE gel electrophoresis using 9 % poly-acrylamide gels, blotted to nitrocellulose membranes (Thermofisher Scientific), and probed with the following primary antibodies: anti-Vinculin (1:5000, Sigma-Aldrich# V9131, Dorset, UK), anti-CD19 (1:1000, Cell Signaling #3574, Danvers, MA), anti-Cyclin D2 (1:1000, Cell Signaling #3741), anti-Cyclin D1 (1:1000, Cell Signaling #55506) at 4°C overnight. Antibodies were diluted in 5 % BSA in TBS-T.

For visualization, the membranes were incubated with IRDye800CW goat-anti-rabbit or goat-anti-mouse IgG antibodies (1:5000, LI-COR, #925-32211 and 925-32210) for 1 h at room temperature and then scanned with an Odyssey DLX Imaging System (LiCor). PageRuler Plus Prestained Protein Ladder (Thermofisher Scientific, #26620) was used as molecular weight marker. Protein levels were quantified by Image J (NIH, MD, USA).

**Statistical analyses**

Data analyses were performed using GraphPad Prism 9.4.1 (GraphPad Software, La Jolla, USA). Unpaired or Paired Student’s t-test was used to compare two groups in all experiments as indicated. Shapiro-Wilk test was used to test for normal distribution. Statistical annotations were denoted with asterisks as follows: ****P <0.0001, ***P < 0.001, **P < 0.01, *P < 0.05, and not significant (ns) P > 0.05.

**Supplementary references**

1. Mangolini M, Maiques-Diaz A, Charalampopoulou S, et al. Viral transduction of primary human lymphoma B cells reveals mechanisms of NOTCH-mediated immune escape. Nat Commun. 2022; 13 (1):6220.
2. Wu CM, Roth TL, Baglaenko Y, et al. Genetic engineering in primary human B cells with CRISPR-Cas9 ribonucleoproteins. J Immunol Methods. 2018;457:33-40.
3. Brinkman EK, Chen T, Amendola M, et al. Easy quantitative assessment of genome editing by sequence trace decomposition. Nucleic Acids Res. 2014;42(22):e168.
